# Wheat Extract Oil (WEO) Attenuates UVB-Induced Photoaging via Collagen Synthesis in Human Keratinocytes and Hairless Mice

**DOI:** 10.3390/nu12020300

**Published:** 2020-01-22

**Authors:** Dong Ju Son, Jae Chul Jung, Yong Min Choi, Hyeon Yeol Ryu, Somin Lee, Barbara A. Davis

**Affiliations:** 1 College of Pharmacy and Medical Research Center, Chungbuk National University, Osongsaengmyeong 1-ro 194-21, Osong-eup, Heungduk-gu, Cheongju, Chungbuk 28160, Korea; sondj1@chungbuk.ac.kr; 2 Life Science Research Institute, NOVAREX Co., Ltd., Ochang, Cheongju 28126, Korea; jcjung@novarex.co.kr (J.C.J.); ymchoi81@novarex.co.kr (Y.M.C.); 3 Korea Conformity Laboratories, 8, Gaetbeol-ro 145 beon-gil, Yeonsu-gu, Incheon 21999, Korea; rhyckato98@kcl.re.kr (H.Y.R.); somin14@kcl.re.kr (S.L.); 4 PLT Health Solutions Inc., 119 Headquarters Plaza, Morristown, NJ 07960, USA

**Keywords:** collagen, hydration, polar lipids, wheat (*Triticum vulgare/aestivum*), photoaging, hairless mice

## Abstract

The efficacy of wheat extract oil (WEO), standardized to glucosylceramides, for protecting against ultraviolet B (UVB)-induced damage of skin barrier function was assessed using the SHK-1 hairless mouse model and two human skin cell lines, namely, CCD-986sk and HeCaT. The ability of repeated oral administration of 30, 60, and 120 mg of WEO/kg/day for 12 weeks to prevent skin damage of SKH-1 hairless mice induced by UVB irradiation was evaluated. The results demonstrated that UVB-induced water evaporation (transepidermal water loss, TEWL) was significantly decreased by WEO. Similarly, UVB-induced losses in moisture and skin elasticity were improved by WEO supplementation. WEO attenuated the tissue procollagen type I, hyaluronic acid (HA), and ceramide reductions induced by UVB treatment as well. Collagen concentrations in skin tissue were increased in the WEO-treated mice, while UVB-induced epidermal thickening was reduced. In vitro studies using HeCaT human keratinocytes confirmed increased HA and collagen synthesis in response to WEO treatment. This may occur via WEO suppression of matrix metalloproteinase-1 (MMP-1), since its induction by UVB treatment was diminished in treated CCD-986sk cells. Oral administration of WEO improves skin barrier function in UVB-irradiated mice by attenuating damage typically observed in photoaging. This research further clarifies the clinical benefits previously observed by dietary WEO consumption.

## 1. Introduction

Skin has a critical barrier function, protecting the body from external elements and pathogenic microorganisms; as such, the integrity of this organ is vital to health [1]. With aging, structural changes that can disrupt the integrity of the skin barrier are observed. One clinical manifestation of these changes is dry skin caused by transepidermal water loss across a thinning epidermal layer [1,2]. Environment, age, and physical condition can all impact signs and symptoms of dry skin, and while both intrinsic and extrinsic factors are responsible for skin aging, ultraviolet B (UVB) light exposure triggers responses leading to photoaging of the skin [2].

The three major layers of the skin include the epidermis, dermis, and hypodermis. The outermost layer, the epidermis, provides a moisture barrier and gives skin its tone [3]. The outer layer of the epidermis, or stratum corneum, is composed of lipids and keratinocytes. Here, there are three major lipids: cholesterol, fatty acids, and ceramides. As a key structural component, ceramides represent 35–40% of the lipid “cement” that ensures cell cohesion within this layer and helps to maintain moisture [4,5,6,7].

Various environmental factors and conditions may damage the barrier of the stratum corneum, causing excessive dryness of the skin [8]. Ceramides are sphingolipids that serve a critical role in moisturizing and other epidermal barrier functions [9]. A lack of ceramide in the skin results in a nonfunctional hydrolipidic barrier [10], and applying ceramides to dry skin improves hydration [11,12]. The content of ceramide in the skin has been shown to decrease with age [7,13,14,15] and, as such, represents a prime target for dietary ingredients aimed at enhancing skin health.

Ceramides are produced endogenously but can be obtained from diet as well. It has been demonstrated that dietary sphingolipids are hydrolyzed in the small intestine, transported across the brush border membrane, and enter the circulation via chylomicrons [16,17,18].

The relationship between nutrition and the quality of the skin is strongly supported by studies that show skin damage such as dermatitis and skin discoloration caused by nutrient deficiency [19]. Therefore, some dietary ingredients play an active role in the physiology of the skin and can affect aging. Several studies have shown the benefits of plant extracts in preventing and improving skin aging [20,21].

Ceratiq^®^, a lipid extracted from wheat grain (*Triticum vulgare/aestivum*), is a plant extract rich in polar lipids used as a nutritional supplement for skin moisturizing [22]. As with many food extracts, the composition of wheat extract oil (WEO) is complex. A recent study partially characterized the composition of WEO as containing predominantly phospholipids, glycolipids, and sphingolipids [23]. The extract is standardized to glycosylceramides (≥2%) and digalactosyldiacylglycerol (DGDG) (≥15%) to ensure consistency among batches.

Both oil and powder forms of WEO show significant improvement in skin hydration [24,25]. A recent clinical study demonstrated that endpoints related to moisturizing, thickness, roughness, and erythema significantly improved after 3 months of administration of WEO in healthy women [24]. However, a detailed study of the mechanism(s) through which WEO exerts its skin benefits has not been conducted.

The present report describes studies to determine the efficacy of WEO in modulating the effects of UVB damage on HaCaT and CCD-986sk cells and in hairless mice. Also presented here are data that help to describe the underlying mechanism of WEO skin benefits.

## 2. Material and Method

### 2.1. Preparation of WEO

WEO prepared from the endosperm of wheat grain (*T. vulgare/aestivum*) and commercially available as Lipowheat^®^ (Robertet, Grasse, France) or Ceratiq^®^ (distributed by PLT Health Solutions) was obtained according to a patented manufacturing process [25]. This extract is approved as a food supplement by the DGCCRF (Direction Générale de la Consommation, de la Concurrence et de la Répression des Fraudes) in France and complies with manufacturing process and residual solvent regulations established by the European Union. Ceratiq/Lipowheat is also licensed in Canada (NPN #80069569) and has New Dietary Ingredient (NDI) status from the Food & Drug Administration (FDA) in the United States.

### 2.2. Test Material Preparation

The test material WEO was diluted to 120, 60, and 30 mg/kg with an excipient (corn oil, Sigma Aldrich) for high, medium, and low doses, respectively. Hyaluronic acid (HA), obtained from SK Bioland (Ansan, Gyeongggi, Korea), was diluted to 60 mg/kg with water and served as a positive control. Test materials were kept at room temperature.

### 2.3. In Vivo Experiments

#### 2.3.1. Experimental Animals and UVB Irradiation

Healthy, 6-week-old SKH-1 hairless mice, purchased from OrientBio (Jungwon-gu, Seongnam-si, Gyeonggi-do, Korea), were selected for use in this study following a 5-day acclimatization period. Mice were maintained in a climate- and light-controlled (22 ± 3 °C and a relative humidity of 50% ± 20%, 12 h light/dark cycle) barrier facility with free access to food and water (Teklad Certified Irradiated Global 18% Protein Rodent Diet, ENVIGO Co. Ltd., Madison, WI). Animal testing and maintenance were conducted under conditions approved by the Institutional Animal Care and Use Committee of Korea Conformity Laboratories (IA18-00939). Mice were randomly assigned into six groups (*n* = 8/group, Table 1): normal control (NC) group, UVB group, UVB + WEO 30 group, UVB + WEO 60, UVB + WEO 120, and UVB + HA group. Test materials were administered orally via disposable oral gavage needles in the morning daily for 12 weeks.

An ultraviolet irradiation system (LK3500) fitted with a UVB lamp (5 Sankyo Denki G5T5E lamps, Sankyo Denki Co., Kanawaga, Japan) was used to induce skin damage to simulate photoaging. UV irradiation intensity was measured using a UV radiometer (HD2101.1, Luckyscientech, Bucheon, Gyeonggi, Korea). Through preliminary testing, we determined the amount of ultraviolet irradiation required for a minimal erythemal dose (MED) to be 80 mJ/cm^2^. The 12-week schedule of UVB dosing included one MED the first week, increasing by one MED per week until reaching four MEDs, which was maintained for the remainder of the study period. Observations were conducted three times per week (Monday, Wednesday, and Friday) for 12 weeks.

#### 2.3.2. Skin Hydration and Transepidermal Water Loss (TEWL) Measurement

Hydration of dorsal skin was assessed using a CM825 Corneometer (Courage & Khazaka Electronics, Cologne, Germany) prior to, then at 1, 2, 5, 8, 10, and 12 weeks after UVB exposure to determine epidermal water content. A TM 300 Tewameter (Courage & Khazaka Electronics, Cologne, Germany) was used to measure transepidermal water loss at the same timepoints prior to and following UVB irradiation used for analysis by corneometry.

#### 2.3.3. Skin Elasticity and Wrinkle Measurement

Skin elasticity of dorsal skin samples was measured, at the same timepoints used for corneometry and TEWL assessments, using a Cutometer MPA580 (Courage & Khazaka Electronics, Cologne, Germany). At week 12, a Visioscan VC98 (Courage & Khazaka Electronics, Cologne, Germany) was used to take a photograph of the epidermis and images were analyzed to evaluate the presence of wrinkles. The results are expressed as a percentage of wrinkles in the skin’s epidermis before the autopsy.2.3.4. Preparation and Analysis of Skin Tissue

At the end of the study, the dorsal skin was collected and placed dermis side down in a 1:1 mixture of Hank’s balanced salt solution (HBSS) and dispase solution (grade II, 2.4 unit/mL) at 4 °C for a period of 18 h to separate the epidermis. To determine procollagen type I and HA, the tissue was pulverized in protein lysis buffer and analyzed via ELISA kits.

#### 2.3.4. Ceramide Content of Skin Tissue

For skin ceramide analysis, 400 μL of a 2:1 (v/v) chloroform:methanol solution was added to epidermal tissue, taken using an 8 mm biopsy punch, and centrifuged for 5 min at 3000 rpm. The resulting pellet was analyzed for ceramide using LC/MS/MS.

Briefly, 2 mL of methanol was added to samples and vortexed for 30 s to extract lipids. Samples were analyzed using ultra-high-performance liquid chromatography (1290 Infinity, Agilent, Santa Clara, CA, USA) coupled to a triple quadrupole mass spectrometer (6460 Triple Quadruple, Agilent, Santa Clara, CA, USA). Analytes were separated using a C18 column (Poroshell 120 EC-C18, 2.7 µm, 21.1 × 100 mm; Agilent, Santa Clara, CA, USA) with 0.1% formic acid in acetonitrile at a flow rate of 0.4 µL/min. Analytes were fragmented using N_2_ as a collision gas and monitored in positive ion mode by multiple reaction monitoring (MRM).

#### 2.3.5. Histopathological Assessment of Skin Tissue

Following autopsy, histopathological examination was performed on dorsal skin tissue using hematoxylin and eosin (H&E) and Masson’s trichrome stain. Stained tissues were photographed using AxioVision SE64 (ZEISS, Oberkochen, Germany) and analyzed using Carl Zeiss AxioVision SE64 Rel. 4 software to measure epidermal thickness.

### 2.4. Cell Culture Experiments

#### 2.4.1. HaCaT Cell Culture and Viability

HaCaT cells (obtained from CLS Cell Line Service GmbH, Eppelheim, Germany) were maintained at 37 °C under 5% CO_2_ (Forma Scientific, Waltham, MA, USA) in DMEM containing 1% P/S (100 units/mL penicillin and 100 µg/mL streptomycin) and 10% fetal bovine serum (FBS; Gibco, Gaithersburg, MD, USA). The cells were maintained in 100 mm cell culture dishes and used when they reached 80% density.

Cell viability was assessed using CCK-8 (Dojindo Molecular Technologies, Inc., Rockville, MD, USA, Lot No. KL709). Briefly, HaCaT cells (5 × 10^4^ cells/well) were cultured in 96-well culture plates for 24 h. After removing the culture medium, extracts were cultured with WEO at concentrations of 28.20–900 ug/mL and incubated for 24 h, after which 10 μL of CCK-8 solution was added to each. Following a 2 h of incubation, absorbance was measured on a microplate reader (Molecular Devices, SoftMax Pro5, San Jose, CA, USA) at a wave length of 450 nm. Cell viability based on the measured optical density (OD) was determined according to the following calculation: % viable cells = (OD of test group/OD of negative control group) ×100.

#### 2.4.2. CCD-986sk Cell Culture and Viability

CCD-986sk cells (Korean Cell Line Bank, KCLB No. 21947), human dermal fibroblast cell line, were maintained in an incubator at 37 °C under 5% CO_2_ (Forma Scientific, Waltham, MA, USA) using IMDM with 1% P/S in 10% FBS (Gibco, Gaithersburg, MD, USA). The cells were maintained in 100 mm cell culture dishes and used when they reached a density of 80%. Cell viability in the presence of WEO was assessed using CCK-8 as described above.

#### 2.4.3. Collagen Formation Test

CCD-986sk cells were used to determine the effect of WEO treatment on the synthesis of procollagen type I. For these experiments, cells were plated on 48-well plates at 5 × 10^4^ cells/well and incubated for 24 h. Following this, culture media was removed and 5 ng/mL of TGF-β was added to the WEO and positive control cells and incubated for an additional 24 h. The supernatant was recovered from each well and used for procollagen type I analysis using the procollagen type I C-Peptide ElAkit according to the manufacturer’s directions (MK101, Takara-Bio Inc., Kusatsu, Japan). The sensitivity for this kit is 10 ng/mL; analyzed samples fell within the 10–640 ng/mL standard curve.

#### 2.4.4. Hyaluronic Acid Formation Test

HA secretion was measured in the human keratinocyte (HaCaT) cell line in response to UVB irradiation and incubation with WEO. HaCaT cells were plated (2 × 10^5^ cells/well) on a 24-well plate and incubated for 24 h. Following this, culture media was replaced with media containing various concentrations of WEO (0, 50, 100, 200, and 400 μg/mL) for 24 h. Cells were then washed with PBS, media was replaced with FBS-free media, and cells were treated with UVB (3 J/cm^2^) and incubated for an additional 24 h. The supernatant was recovered from each well and the total amount of HA was measured according to the manufacturer’s instructions using an HA quantitative test kit (HAE-163, Corgenix, Broomfield, CO, USA). The sensitivity for this kit is 20 ng/mL; analyzed samples fell within the 0–800 ng/mL standard curve.

#### 2.4.5. Matrix Metalloproteinase-1 (MMP-1) Formation Test

CCD-986sk cells were used to assess MMP-1 production in response to UVB irradiation in the presence of WEO at concentrations of 0, 50, 100, 200, and 400 mg/mL. Cells were plated (5 × 10^4^ cells/well) on a 24-well plate and maintained for 24 h. Following this, cells were washed with PBS and media was replaced with FBS-free media and irradiated with UVB (40 mJ/cm^2^). The recovered supernatant from each well was analyzed for MMP-1 using the human MMP-1 ELISA kit (Abcam, Cambridge, MA, USA) according to the manufacturer’s instructions. The sensitivity for this kit is <8 pg/mL; analyzed samples fell within the 24.60–180,000 pg/mL range of the standard curve.

#### 2.4.6. RNA Isolation and Quantitative RT-PCR

The NucleoSpin RNA II kit (Macherey-Nagel, Duren, Germany) was used to isolate total RNA and the NanoDrop 2000c (Thermo Scientific, Waltham, MA, USA) was used to determine the RNA concentrations. Total RNA (500 ng) was reverse-transcribed to cDNA with the REVERTRA Ace qPCR RT Master Mix (TOYOBO, Osaka, Japan). PCR was performed using the SYBR Green PCR Master Mix (TOYOBO). The primers used in this study were as follows: MMP-1, F: 5′-GGG GCT TTG ATG TAC CCT AGC-3′ A, R: 5′-TGT CAC ACG CTT TTG GGG TTT-3′; β-actin, F: 5′-ACG TCG ACA TCC GCA AAG ACC TC-3′, R: 5′-TGA TCT CCT TCT GCA TCC GGT CA-3′.

### 2.5. Statistics

All results are expressed as mean ± standard deviation (SD) and were analyzed using a one-way analysis of variance (ANOVA) followed by the Newman–Keuls multiple range test (parametric). Statistical significance was set at *p* < 0.05. The statistical analysis program used was SPSS 12.0 K (SPSS, Chicago, IL, USA.).

## 3. Results

### 3.1. Skin Hydration and Transepidermal Water Loss

**Figure 1:**
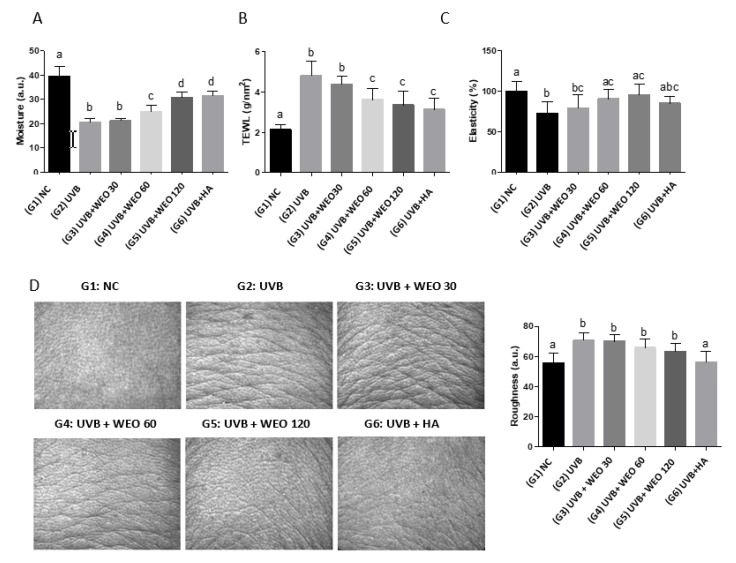
WEO-induced changes in skin surface condition at week 12. (**A**) Skin moisture measurement by Corneometer CM825. (**B**) Transepidermal water loss measurement by Tewameter TM 300. (**C**) Skin elasticity measurement by Cutometer MPA580. (**D**) Roughness measurement by Visioscan VC98. Values with different letters are significantly different by analysis of variance (ANOVA) followed by the Newman– Keuls multiple range test (*p* < 0.05). NC: normal control, UVB: Ultraviolet, WEO: wheat extract oil, HA: Hyaluronic acid.

Stratum corneum hydration and TEWL were evaluated to determine the effect of WEO treatment on skin barrier function. While hydration of skin was reduced in response to UVB treatment, mice supplemented with WEO started to show better hydration of the stratum corneum compared with UVB-exposed control mice beginning at 8 weeks (data not shown, *p* > 0.05). Moisture in all treated groups remained lower than in the NC group; however, the UVB + WEO 120 group was shown to have the most effective WEO dose, achieving protective benefits similar to the UVB + HA positive control. Statistically significant increases in stratum corneum water content were observed in the WEO-supplemented groups compared with the UVB group by week 10 (data not shown, *p* < 0.01) and continued throughout the study (week 12 data shown in Figure 1A, *p* < 0.05). No statistically significant differences were observed between the UVB + WEO 30 and UVB groups.

Continuous UVB exposure increased water evaporation in the control group compared with WEO-treated mice as expected (data not shown). While moisture loss following WEO administration tended to decrease from the eighth week compared with the UVB-exposed control group, the water evaporation rate was reduced significantly in the UVB + WEO 60 and 120 groups relative to the UVB-exposed control group by week 12 (Figure 1B, *p* < 0.05).

### 3.2. Skin Elasticity and Wrinkles in the Dorsal Epidermis

As shown in Figure 1C, WEO administration at both the 60 and 120 mg/kg/day doses resulted in statistically significant increases in skin elasticity relative to the UVB control (*p* < 0.05). While UVB irradiation increased wrinkles in the epidermis of all treated mice (Figure 1D), WEO administration showed a tendency toward improvement in both the 60 and 120 mg/kg/day dose groups, although this did not achieve statistical significance compared with the UVB control (*p* > 0.05).

### 3.3. Procollagen Type I, Hyaluronic Acid, Ceramide, and Collagen in Skin of Hairless Mice Improved with WEO Administration

**Figure 2:**
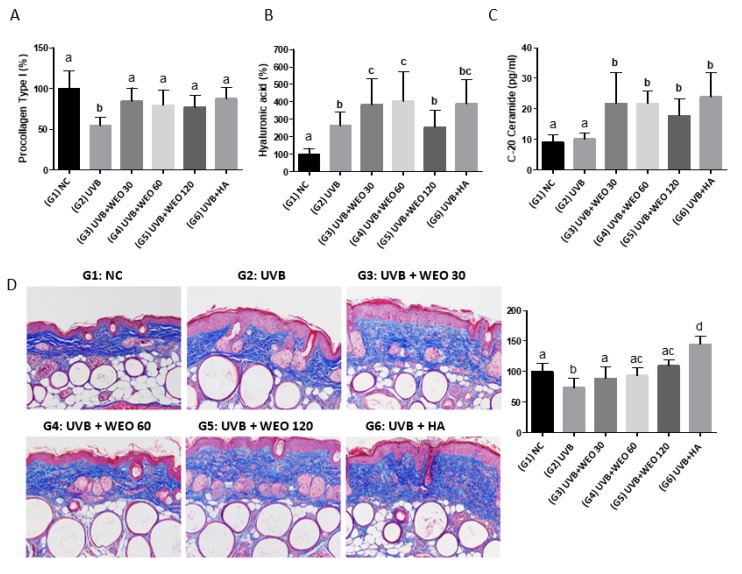
Effect of WEO on skin tissue components at 12 weeks. (**A**) Procollagen type I measurement by ELISA. (**B**) Hyaluronic acid measurement by ELISA. (**C**) Ceramide measurement by LC/MS/MS. (**D**) Collagen measurement by Masson’s trichrome stain. Values with different letters are significantly different by ANOVA followed by the Newman–Keuls multiple range test (*p* < 0.05). NC: normal control, UVB: Ultraviolet, WEO: wheat extract oil, HA: Hyaluronic acid.

Collagen is synthesized in precursor form as procollagen, with type I collagen being the main form [26]. In this study, procollagen type I decreased in response to UVB irradiation. Figure 2A shows this loss was prevented by the administration of WEO at all three concentrations (30, 60, and 120 mg/kg/day) relative to the UVB-treated control group (*p* < 0.05).

HA may initially increase in the dermal layer in response to UVB exposure as a skin defense mechanism [27,28,29]. In this study, the concentration of HA in the UVB + WEO 30 and 60 groups increased significantly (*p* < 0.05) compared with the UVB-treated group but not in the UVB + WEO 120 group (Figure 2B).

### 3.4. Ceramide Measurement

Although some studies [8,9] have shown continuous UVB exposure alters the ceramide content of the epidermis, we did not observe this in the present study. However, as is shown in Figure 2C, all doses of WEO extract resulted in statistically significant increases in skin ceramide content relative to the normal and UVB-treated control animals (*p* < 0.05).

Collagen density decreased in response to UVB irradiation in the untreated group. Both 60 and 120 mg/kg/day WEO supplementation significantly increased (*p* < 0.5) collagen levels relative to UVB-treated mice (Figure 2D).

### 3.5. Epidermal Thickness

**Figure 3:**
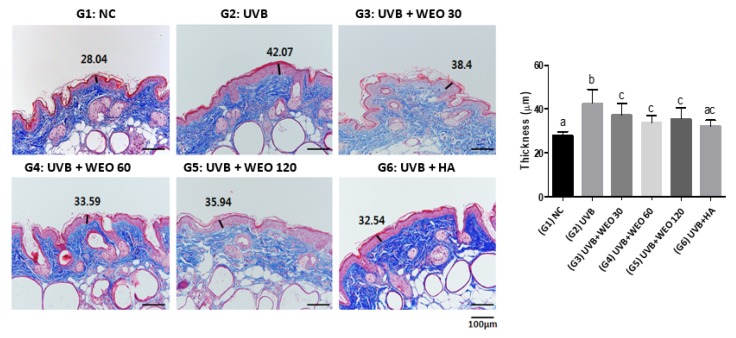
Epidermis thickness measurement (Masson’s trichome stain) at week 12. Values with different letters are significantly different by ANOVA followed by the Newman–Keuls multiple range test (*p* < 0.05). NC: normal control, UVB: Ultraviolet, WEO: wheat extract oil, HA: Hyaluronic acid.

Masson’s trichrome staining (Figure 3) revealed that the epidermal thickness, increased by UVB treatment, was significantly lower in all three groups administered WEO relative to the UVB group (*p* < 0.05).

### 3.6. In Vitro Determination of WEO on Procollagen, Hyaluronic Acid, and Matrix Metalloproteinase-1 Synthesis

**Figure 4:**
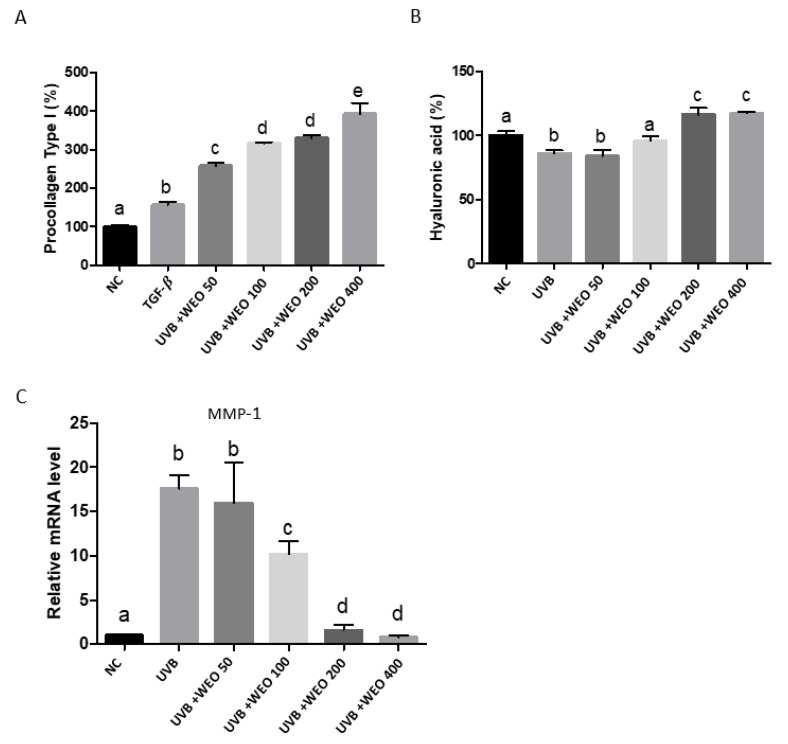
Changes in skin-damage-related factors in human skin cell lines. (**A**) Procollagen synthetic enhancement measurement in CCD-986sk cells by microplate reader. (**B**) Hyaluronic acid measurement in HaCaT cells. (**C**) Measurement of matrix metalloproteinase-1 (MMP-1) mRNA expression in CCD-986sk cells by qRT-PCR. Values with different letters are significantly different by ANOVA followed by the Newman–Keuls multiple range test (*p* < 0.05). NC: normal control, UVB: Ultraviolet, WEO: wheat extract oil, HA: Hyaluronic acid.

The ability of WEO to modulate procollagen synthesis was evaluated in the human dermal fibroblast cell line CCD-986sk, using TGF-β as a positive control. As shown in Figure 4A, the addition of WEO to culture media statistically significantly increased procollagen synthesis over untreated and TGF-β-treated cells at all doses tested (*p* < 0.05).

UVB irradiation dramatically increased MMP-1 mRNA levels in CCD-986-sk cells compared with untreated cells (Figure 4C, *p* < 0.05). This increase was attenuated by WEO treatment of cells at 100, 200, and 400 µg/mL (*p* < 0.05).

HaCaT cells, cultured with increasing concentrations of WEO, were subjected to UVB treatment, after which HA was measured (Figure 4B). As expected, irradiation decreased HA relative to untreated control cells. This effect was reversed by WEO in a dose-dependent manner (*p* < 0.01).

## 4. Discussion

WEO is a source of phytoceramides, including glucosylceramides [24]. In recent clinical studies, participants consuming WEO have seen improvements in skin hydration and the appearance of wrinkles [24,30]. Despite this, an analysis of the underlying mechanism(s) through which WEO exerts its effects has not been reported previously.

In this study, the effects of 12 weeks of daily oral WEO administration on UVB-induced skin damage in SKH-1 hairless mice were evaluated. Specifically, skin surface (moisture level, moisture evaporation, elasticity, and wrinkles), skin tissue composition (procollagen type I, hyaluronic acid, and ceramide), and histopathological (collagen and the thickness of epidermis) responses to WEO administration were assessed, with HA treatment used as a positive control.

Continuous exposure to UV rays reduceds the amount of skin moisture level as a result of damage to skin epidermis and dermis tissue. This results in loss of skin elasticity and the creation of dry and rough skin [31,32]. In the current study, the moisture content of the skin increased statistically significantly relative to the UVB-treated control group (Figure 1A, *p* < 0.5) as a result of WEO administration, whereas water loss was reduced relative to the UVB-treated control group (Figure 1B, *p* < 0.05). Further, while the UVB-treated groups supplemented with WEO did not achieve the moisture of the NC group, moisture levels in the highest dosage group (UVB + WEO 120) did reach the positive control moisture levels. Corresponding to this improvement in skin hydration, we observed a statistically significant increase (*p* < 0.05) in skin elasticity relative to the UVB-treated group but not the NC group, with WEO intakes of 60 and 120 mg/kg/day. Based on these results, it can be concluded that daily WEO administration lessens the damaging effect of UVB irradiation on the skin of hairless mice.

The main components of the stratum corneum are ceramide, cholesterol, and free fatty acids [4,5]. These lipids, especially ceramides, play a key role in maintaining skin hydration, ensuring a patent skin barrier against external insults and pathogens. The HA distributed in the dermis is a type of glycosaminoglycan (GAG) with excellent moisture-binding properties that helps to maintain skin hydration [33]. Procollagen type I also plays a role in supporting moisture in the dermis to prevent wrinkles and maintain skin elasticity [34]. To understand the role of WEO in protection against UVB damage, we evaluated changes in the concentration of these three compounds in skin samples from hairless mice.

Compared with the UVB-treated control, both ceramide and procollagen type I concentrations increased significantly in the UVB + WEO 30, 60, and 120 dose groups, while significant increases in HA were observed in the UVB + WEO 30 and 60 dose groups. Maintenance and upregulation of these protective factors suggest they play important roles in the moisture and skin integrity benefits observed with WEO administration in this study. That the 120 mg/kg WEO dose did not show a similar increase in HA is presently unexplained. However, HA increase in response to WEO supplementation was confirmed in HaCaT cells. To our knowledge, this is the first work demonstrating a benefit of WEO on HA skin concentrations. Follow-up work to more fully understand this relationship is warranted.

UV exposure results in incomplete keratinization, producing a thickened epidermal layer with reduced moisture and collagen content [35]. In this study, histopathologic analysis demonstrated that the thickness of the epidermis was significantly decreased in the WEO administration groups, whereas the amount of collagen increased significantly relative to UVB-treated controls.

Oral administration of WEO ameliorated the destructive effects of UVB treatment as demonstrated by increased hydration and elasticity, along with decreased epidermal thickening. These physical effects were accompanied by an attenuation of the UVB-induced upregulation of the collagen-degrading enzyme MMP-1. Procollagen type I, collagen, and HA were also favorably impacted by WEO administration to UVB-treated mice and appear to play a role in the skin-preserving mechanism of this ingredient.

To further define the role of WEO in the prevention of skin photoaging, we used human keratinocyte and fibroblast cell lines. Incubation of CCD-986sk cells with WEO resulted in a concentration-dependent increase in procollagen synthesis. Additionally, while UVB treatment of this cell line increased MMP-1 expression, WEO supplementation decreased this effect in a concentration-dependent manner. Collagen is synthesized in the form of a precursor (procollagen) [36], and as a major structural protein present in the dermal layer, it is critical for skin elasticity. Eighty to ninety percent of skin collagen is type I collagen and is essential for preventing skin damage from UV rays, maintaining the moisture balance to prevent wrinkles and preserve the elasticity of the skin [37]. MMP-1, as a collagenase-specific protease, has been shown to facilitate the decomposition of collagen and to reduce elasticity [38,39]. Synthesis of matrix metalloproteinases, including MMP-1, is upregulated when the mitogen-activated protein kinases (MAPKs) ERK, JNK, and p38 are activated by an external stimulus and is therefore an attractive target for development of agents to delay photoaging [39]. Decreasing degradation of collagen via downregulation of MMP-1 may prevent skin aging and wrinkles. This study has demonstrated that WEO not only upregulates procollagen synthesis but suppresses MMP-1 induction by UVB irradiation, thereby identifying the mechanism that results in increased collagen synthesis following WEO treatment.

Furthermore, we showed that the UVB-induced decrease in hyaluronic acid synthesis was attenuated, with HA concentrations increasing in a dose-dependent manner, by WEO pretreatment of HaCaT cells. Hyaluronic acid, an important component of the extracellular matrix (ECM) of the dermis, is a GAG, which is a large polysaccharide capable of absorbing up to 1000× its weight in water. GAGs are a complex of repeated units of disaccharides alternately combined with N-acetyl-D-glucosamine and D-glucuronic acid, which is a polymer compound with a molecular weight of between 200,000 and 400,000 units [33]. They are involved in maintaining tissue moisture, intercellular spacing, cell division, cell differentiation, cell growth, and tissue healing responses through the storage of moisture. Skin HA is reported to decrease with age, and because of its role in moisture retention, this reduction is considered to be one of the direct causes of increased wrinkles, reduced elasticity, and decreased moisture content in the skin observed in aging skin [40,41]. Our study shows that HA is increased by WEO treatment in both hairless mice and HaCaT cells and likely plays an important role in the hydration benefits observed for WEO.

Continuous exposure to UV rays results in photoaging, characterized by reduced hydration and increased wrinkles, which results from the destruction of key components of the skin epidermis and dermis [35]. Evaluation of dietary interventions to protect skin and reduce the effects of aging is of considerable interest. In the current study, a SKH-1 hairless mouse model was used to evaluate the effect of repeated oral administration of WEO on skin damage induced by 12 weeks of UVB irradiation. We observed increased skin hydration, decreased moisture loss, increased skin elasticity, and a reduction in visible wrinkles. The accompanying changes in skin structural components, including increased ceramide, HA, and procollagen type I, along with a decrease in epidermal thickness, all highlight the mechanisms by which WEO protects against UVB photoaging in this animal model. These results were further confirmed in two human cell lines and also demonstrated a reduction in the collagen-degrading enzyme MMP-1. Therefore, the results of this study indicate that WEO has a protective effect against damage to skin (moisture loss and wrinkles) caused by continuous exposure to UVB rays.

These findings are consistent with those of previous clinical studies showing the beneficial effects of WEO on the skin health of women and help to define the mechanisms through which this ingredient enhances skin hydration and reduces the signs of wrinkles.

## Figures and Tables

**Table 1:** Treatment groups.

Group		Volume (mg/kg)
**G1**	Normal control group	0
**G2**	UVB control group	0
**G3**	UVB + WEO	30
**G4**		60
**G5**		120
**G6**	UVB + Positive control (HA)	60

Abbreviations: UVB—ultraviolet B; WEO—wheat extract oil; HA—hyaluronic acid.
